# Engineering Complex Breast Tumor-Stroma Models: TMPyP4-Photodynamic Therapy Is More Effective at the Metastatic Site in Breast Tumors

**DOI:** 10.1021/acsbiomaterials.5c01341

**Published:** 2025-12-29

**Authors:** Salma T. Rafik, Jasmine Ho, Alexander J. MacRobert, Umber Cheema

**Affiliations:** † UCL Centre for 3D Models of Health and Disease, UCL Division of Surgery and Interventional Science, Faculty of Medical Sciences, Charles Bell House, 43-45 Foley Street, 4919 University College London, London W1W 7TY, U.K.; ‡ Department of Clinical Pharmacology, Faculty of Medicine, Alexandria University, Alexandria 21516, Egypt

**Keywords:** breast cancer, tumor-stroma, real-time oxygen monitoring, 3D tumoroids, photodynamic therapy

## Abstract

The breast tumor microenvironment encompasses distinct biophysical, biochemical, and cellular aspects, including a dense extracellular matrix and an array of tumor and stromal cells. The dynamics between tumor cells and their microenvironment can alter tumor behavior and impact treatment responses. Herein, tumor-stroma models (tumoroids) were engineered using dense collagen l to spatially compartmentalize a breast tumor mass in either its primary site (breast) or metastatic site (lung) to test the efficacy of photodynamic therapy (PDT) using a photosensitizer (TMPyP4) as a single treatment and in combination with doxorubicin. For tumoroids with a primary stroma, PDT efficacy was comparable for both MCF-7 and MDA-MB-231. In contrast, MCF-7 tumoroids with a metastatic stroma exhibited a greater treatment response with a 7.2-fold decrease in viability compared to the MCF-7 tumoroids with a primary stroma, whereas only a 1.1-fold decrease was seen for the MDA-MB-231 models. For MDA-MB-231 tumoroids with a primary stroma, combination treatment with PDT and doxorubicin gave the best outcomes. The viability data in the 3D models correlated with noninvasive imaging of hypoxia gradients, where hypoxia became progressively alleviated with increasing treatment efficacy. ln summary, these results highlight the necessity to model the tumor stroma as this can directly impact drug efficacy.

## Graphical abstract

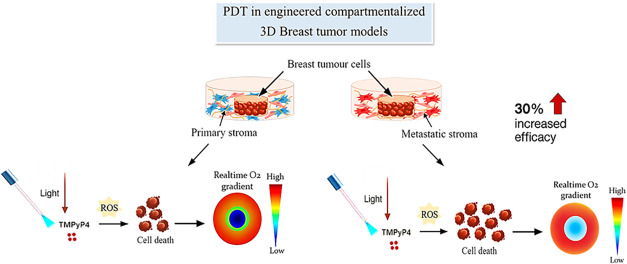

## 1 Background

Breast cancer is the most widespread malignancy and the primary contributor to cancer-related deaths in women across the globe.^1^ It is a heterogeneous disease that can be attributed to the underlying genetics and the tumor microenvironment (TME).^2^ The TME is a dynamic environment that changes over time as disease progresses and comprises cellular components such as tumor cells and nontumor cells including fibroblasts, immune cells, endothelial cells, adipocytes, and mesenchymal stromal cells (MSCs). The TME also contributes a unique biophysical and biochemical microenvironment which includes the oxygenation state, nutrient state, pH gradients, signaling molecules, and extracellular matrix (ECM) components. Stromal cells significantly influence the behavior of cancer cells through direct and indirect means. Stromal cells can remodel the tissue microenvironment directly through the deposition of ECM proteins and matrix-degrading enzymes, but stromal cells also secrete chemokines,^3^ cytokines,^4^ angiogenic and growth factors.^5^ Interactions between stroma and tumor cells, alongside genetic aberrations in tumor cells, determine the growth patterns, phenotype, and metastatic potential of the tumor.^6^ Breast cancer subtypes, including luminal, HER2-enriched, Claudin-Low (CL), and basal-like/triple-negative breast cancer (TNBC), are defined by gene expression of estrogen and progesterone receptor (ER, PR), human epidermal growth factor 2 (HER2), and a cluster of basal genes.^7^ The clinical course and treatment response differ significantly among these subtypes.^8^ For instance, Luminal A tumors generally have the most favorable prognosis,^9^ whereas TNBC (ER^**‑**^, PR^**‑**^, and HER2^**‑**^) is often linked to lower survival rates.^10^ CL tumors exhibit diminished expression of cell-cell adhesion genes, including claudins 3, 4, and 7, occludin, and E-cadherin,^11^ as well as stem cell-like characteristics.^12^

While the existing therapeutic modalities for breast cancer have successfully lowered mortality rates, they still face significant challenges, including systemic side effects due to a lack of tumor-specific accumulation, and the growing problem of drug resistance which complicates treatment outcomes.^13,14^ Drug resistance can be driven by the interplay of several factors, among which are TME components. They play a role in educating cancer cells to escape drug effects through extensive molecular crosstalk. For instance, cancer-associated fibroblasts (CAFs) enhance the aggressiveness and survival of cancer cells through the secretion of growth factors and cytokines, as well as the establishment of a “protective niche” that shields against drugs. Likewise, immune cells contribute to immunosuppression and metastasis.^15^ Adipocytes and MSCs participate in the secretion of factors related to matrix remodeling and tumor survival.^15^ Thus, the TME is now considered to be a novel prognostic and predictive biomarker for breast cancer. Metastases may also display heterogeneous responses to treatment and common sites for metastasis for breast cancer are the brain, bone, and lung.

There is a need for innovative and effective treatment modalities to enhance patient outcomes with one notable alternative being photodynamic therapy (PDT), which has emerged as a minimally invasive therapy for the treatment of diverse cancer types and nonmalignant lesions.^16^ This therapy requires molecular oxygen and a photosensitizer that is activated by specific wavelengths of light, including blue, red, or near-infrared (NIR) light, which triggers the formation of reactive oxygen species (ROS). PDT has received approval for the treatment of tumors, including prostate and esophagus^16^ and provides significant benefits for patients by reducing the necessity for major surgical interventions. PDT carries a low risk of both local and systemic morbidity and is characterized by its tumor selectivity, ease of use, cost-effectiveness, and suitability for repeated applications. A few clinical trials have assessed the efficacy of PDT in breast cancer. The first phase I/lla clinical trial study on PDT treatment of primary breast cancer was conducted by Banerjee et al. (2020) and evaluated verteporfin (BPD)-PDT in a cohort of 12 patients. MRI data correlated with histopathology findings, which revealed that tumor necrosis was augmented with incremental increases in light dose.^17^ Other studies showed that PDT treatment using Photofrin in breast cancer patients with chest wall invasion/recurrences resulted in ∼65% of patients achieving complete responses,^18^ while PDT treatment using m-THPC led to complete responses in all patients.^19^

3D models better replicate the physiological 3D tissue architecture of the TME reflected in biomimetic cell morphological characteristics, cell proliferation, tumor heterogeneity, and drug response compared to monolayer 2D culture.^20^ Herein, we utilized breast tumoroids using dense collagen l as a scaffold for 3D *in vitro* modeling of breast cancer. Spatially compartmentalized dense 3D collagen l gels were engineered to mimic the TME by embedding a tumor mass within a stromal compartment. Tumoroids offer a structural basis for constructing matrix-relevant models, enabling meticulous regulation of the density and composition of the ECM alongside a distinctly defined boundary between tumor and stroma. These models include physical, chemical, and cellular barriers closely replicating the *in vivo* interactions between cancer cells and stromal components.^21^ The stiffness of these scaffolds falls within the range of solid breast tumors, and tumoroids are compatible with monitoring in real-time the formation of hypoxia gradient. The present study aims to investigate the effect of PDT and combined PDT/Chemotherapy in a biomimetic 3D breast tumor model using advanced real-time noninvasive assessment modes.

## 2 Methods

### 2.1 Cell Lines

MDA-MB231 and MCF-7 human breast adenocarcinoma cells were obtained from the European Collection of Authenticated Cell Cultures (ECACC). MCF-7 cells represent luminal A subtype, and MDA-MB-231 cells represent CL tumors. They were cultured using Dulbecco’s Modified Eagle’s Medium/Nutrient Mixture F12 Ham (DMEM/F12) growth medium (Sigma-Aldrich, Dorset, U.K.). Adipose tissue-derived mesenchymal stem cells (MSCs) were acquired from ATCC (American Type Culture Collection, Virginia, United States) and human normal lung fibroblast (HNLF) were purchased from Lonza, Bioscience. Both cell types were cultured in DMEM supplemented with 1g/L d-glucose (Gibco through Thermo Fisher Scientific, Loughborough, U.K.), 10% FBS (Gibco through Thermo Fisher Scientific, Loughborough, U.K.), and 1% penicillin (5000 units/ml)/streptomycin (5000 μg/ml) (Gibco through Thermo Fisher Scientific, Loughborough, U.K.). Human umbilical vein endothelial cells (HUVECs) were acquired from Promocell, Heidelberg, Germany, and were cultured in endothelial growth media supplemented with 10% FBS and 1% penicillin/streptomycin obtained from Promocell, Heidelberg, Germany. All cells were cultured under 5% carbon dioxide (CO2) and 95% atmospheric air at 37 °C, with routine passaging carried out in 2D monolayers. Passage numbers for cells were: HUVECs < 6 passage number, HNLF < 10 passage number, MSCs < 20 passage number, MCF-7 and MDA-MB-231 cells < 50 passage number.

### 2.2 Engineering of 3D Breast Tumor Model

3D tumoroids were constructed using the RAFT protocol (Lonza, Basel, Switzerland), which uses the plastic compression method (Figure 1) to generate physiologically relevant collagen densities.^22^

**Figure 1:**
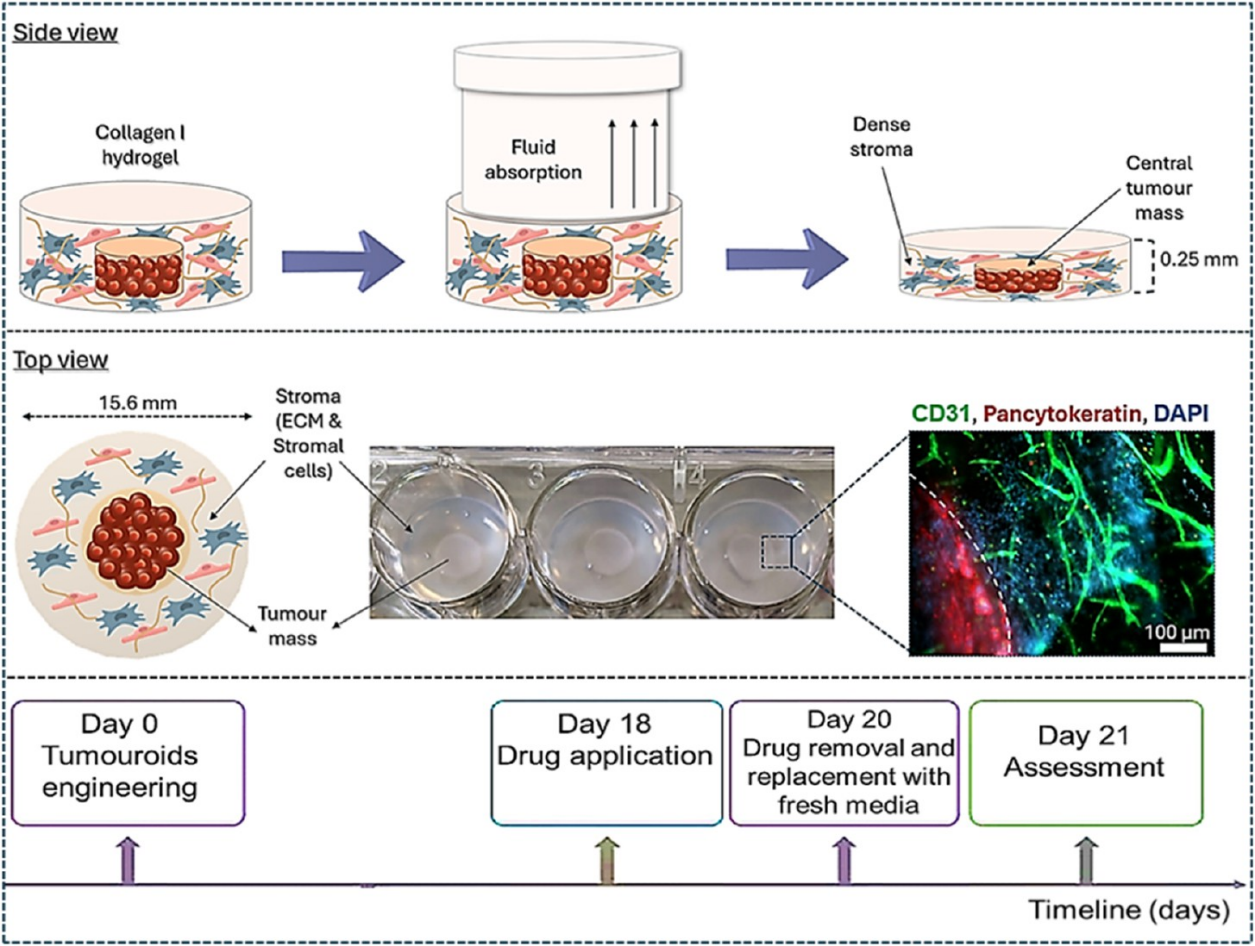
Schematic showing fabrication of breast tumoroids 3D model. Initially, samples are prepared as collagen type I hydrogels with cells incorporated into the matrix. Subsequently, RAFT absorbers are applied to the gels during which fluid is absorbed, leading to the compression of the gel. The resulting gel is a dense collagen matrix with a thickness of approximately 250 μm. A compartmentalized tumoroid consists of a central TM embedded within a surrounding healthy stroma. Dotted line denotes tumor-stroma boundary.

#### 2.2.1 Simple Tumoroids (Tumor Mass)

Tumor mass (TM) of either MDA-MB-231 or MCF-7 at 2 × 10^4^ seeding density was formed by mixing 10xMEM (Gibco through Thermo Fisher Scientific, Loughborough, U.K.), type I collagen 2 mg/ml (First Link, Birmingham, U.K.), 10 μg/ml laminin (Sigma-Aldrich, Dorset, U.K.), neutralizing solution (17% 10 M NaOH (Sigma-Aldrich, Dorset, U.K.) in 1 M HEPES buffer (Gibco through Thermo Fisher Scientific, Loughborough, U.K.) and cells suspended in culture medium. A volume of 240 μL of cell-containing gel mixture was added in 96-well plates (Corning through Thermo Fisher Scientific, Loughborough, U.K.) and incubated at 37 °C for 15 min. Following polymerization, absorbers were placed on top of each gel for 15 min. Following compression, 100 μL of cell culture media was added to each TM. The seeding density was determined by culturing different cell densities within the range of previously published cultures for colorectal cancer (5 × 10^4^ cells per tumor mass) (Pape et al. 2020).^23^ However, following assessment and monitoring of invasion using the breast cancer cell lines, equivalent invasion was measured using 2 × 10^4^ cells, and this was deemed a more sustainable option.

#### 2.2.2 Compartmentalized Tumoroids

The advanced structure of complex tumoroids encompasses a stromal compartment that contains an embedded tumor mass. TM was manufactured as previously described. To model the tumor stroma, a collagen solution was prepared by mixing 10xMEM, type I collagen 2 mg/ml, neutralizing solution (17% 10 M NaOH in 1 M HEPES buffer) with 20 μg/ml of laminin and 2 mg/ml of fibrin (Sigma-Aldrich, Dorset, U.K.) and stromal cell populations. 650 μL of the collagen mix is placed in a 24-well plate (Corning through Thermo Fisher Scientific, Loughborough, U.K.) and first polymerized at 37̊C for 15 min. Then, the TMs were placed on top of the set gels followed by adding a further 650 μL of the collagen mix, and incubated for further 15 min at 37 °C. Once fully polymerized, the tumoroids were plastic-compressed using the 24-well RAFT absorbers at room temperature for 15 min, as shown in Figure 1. Finally, 1 mL of cell culture media was added to each gel. Tumoroids were cultured at 37 °C under 5% CO2 and 95% atmospheric air for 21 days, with 50% of cell culture media replaced every 48 h.

##### 2.2.2.1 Engineering a Primary Stroma Compartment

The stromal compartment is composed of 1 × 10^4^ MSCs and 1 × 10^5^ HUVECs in addition to 20 μg/mL laminin and 2 mg/ml fibrin.

##### 2.2.2.2 Engineering a Metastatic Stroma Compartment

The stromal compartment is composed of 1 × 10^4^ HNLF and 1 × 10^5^ HUVECs in addition to 20 μg/mL laminin and 2 mg/ml fibrin.

Initially, a 1:1 ratio of breast cancer cell lines to stromal cells was attempted because this is a very common ratio utilized by many *in vitro* 3D coculture studies. However, on closer inspection, we observed that the presence of stromal cells with this ratio caused the tumoroids to contract, which made it difficult to conduct specific assessments such as fluorescence imaging. We then tried a 2:1 ratio of breast cancer cell lines to stromal cells (2 × 10^4^ of breast cancer cells and 1 × 10^4^ of stromal cells), which was considered a more convenient option as it maintained both the tumoroids structure stability, cancer invasion, and the development of vascular networks.

#### 2.2.3 Characterization of Stromal Compartments

The primary stromal compartment was comprised of adipose-derived MSCs, and the metastatic stromal compartment was comprised of human normal lung fibroblast (HNLF). Both are cell lines and were acquired from ATCC and Lonza, Bioscience, respectively.

As such, the characterization conducted for these cells focused on how cells behaved *in vitro*. Characterization included observing and quantifying the invasion of both tumor cell lines into compartments containing either MSCs or lung fibroblasts, and showing that invasion is greater in the presence of the lung fibroblast stroma (Supporting 1). This is expected as both tumor cell lines are derived from lung pleural effusions. Furthermore, characterization work demonstrated that MSCs support the aggregation of ECs to fuse and form networks, which is not the case with the lung fibroblasts (Supporting 1). Characterization of the MSCs using flow cytometry analysis to check the “stemness” of the adipose-derived MSCs found positive expression of cell surface markers for CD90, CD73, and CD105 (excess of 93% for all groups) and negative markers for CD19, CD34, CD11b, CD45, and HLA DR (Ho, 2024).^24^

### 2.3 Cell Viability Assay

The cell viability of 2D monolayer cultures and 3D constructs was assessed by measuring ATP levels using CellTiter-Glo 3D Viability Assay (Promega, Southampton, U.K.). At set time points following drug treatment, samples were mixed with CellTiter Glo Reagent with a (1:1) ratio and then subjected to vigorous shaking for 5 min using an orbital shaker followed by 25 min of benchtop incubation at room temperature. 100 μL of the mix was then transferred to a black 96-well plate (Thermo Fisher Scientific, U.K.) in triplicate, and luminescence was measured by the Tecan Infinite Lumi plate reader (Männedorf, Switzerland).

### 2.4 Live-Dead Staining for Fluorescence Imaging

Following drug treatment, the 3D constructs were stained for viability imaging using the Live-dead viability kit (Molecular Probes, Thermo Fisher Scientific, U.K.). Cell culture media was removed, and then 3D constructs were incubated with the Live-dead solution composed of 4 μM Calcein-AM and 2 μM Ethidium homodimer-1 for 40 min at room temperature before imaging with Zeiss AxioObserver with ApoTome.2 instrument and software (Zeiss, Oberkochen, Germany) using green filter (%Ex/Em 495/515 nm) and red filter, respectively (%Ex/Em 495/635 nm).

### 2.5 Immunofluorescence Imaging

The 3D constructs were fixed in 4% formalin (Sigma-Aldrich, Darmstadt, Germany) for 30 min followed by rinsing and storage in phosphate-buffered saline (PBS) (Fisher Scientific, Loughborough, U.K.). Then, the constructs were permeabilized and blocked for 1 h at ambient temperature utilizing a solution composed of 0.3% Triton X-100 and 1% bovine serum albumin (both sourced from Sigma-Aldrich, Dorset, U.K.), dissolved in PBS. Primary antibodies: antipancytokeratin (Genetex, Inc., US) at a dilution of 1:100 and antiplatelet cell adhesion molecule (CD31) mouse JC70/A (Abcam, Cambridge, U.K.) at a dilution of 1:200, were subsequently incubated overnight at 4 °C. Following this incubation, primary antibodies were removed through 3 sequential washes with PBS on a plate shaker. The subsequent day, constructs were exposed to secondary antibodies: 1:1000 antimouse Alexa Fluor 488 IgG H&L (ab150113) and 1:500 antirabbit DyLight 594 (ab96885), both obtained from Abcam (Cambridge, U.K.) for 2.5 h at room temperature. This was followed by 3 consecutive washes in PBS and counterstaining with DAPI, using NucBlue (Invitrogen through Thermo Fisher Scientific, Loughborough, U.K.) 20 min before imaging.

### 2.6 Assessment of Oxygen Gradient Level in 3D Constructs

Spatio-temporal oxygen gradient levels within the 3D constructs were examined using the minimally invasive oxygen mapping technique provided by a VisiSens TD MIC system, which relies on collecting luminescence signals from an oxygen-sensitive luminescent foil placed in contact with the construct. The spatially resolved luminescence signal is captured by quantitative 2D imaging. The 3D constructs were placed on top of the oxygen sensor foils glued to the bottom of a 24-well imaging sensor plate ISP24-RPSu4 (PreSens Precision Sensing GmbH, Regensburg, Germany). 1 mL of cell culture media was added to each well. Then, the 24-well plate was incubated at 37 °C, and a 1 h time series of measurements was set for recording signals over a period of 24 h. For calibration, sodium sulfite 1% (wt/V) solution was used for the 0% oxygen baseline, and air-equilibrated distilled water was used for the 100% calibration marker, where 100% corresponds to air oxygen saturation. VisiSens ScientifiCal Software Version VS 1.0.1.5 Plugins was used to analyze the data by selecting the tumor mass as a region of interest, then a graph of the analyte versus time was plotted. Upon reaching a steady state level, an average oxygen percentage was calculated and analyzed via the GraphPad Prism 9 software.

### 2.7 Evaluation of Endothelial Networks Complexity

Zeiss AxioObserver equipped with the ApoTome.2 instrument and software (Zeiss, Oberkochen, Germany) was used for imaging 3D constructs. Four images were captured at 2.5× magnification, positioned at the 12, 3, 6, and 9 o’clock orientations on the same focal plane, and subsequently uploaded to Fiji (NIH open-source software). To characterize the complexity of the endothelial networks present within the stromal compartment, images were analyzed for the number of loops, junctions, branches, and total branch lengths with statistical significance determined using GraphPad Prism 9 software.

### 2.8 Experiments in 2D

#### 2.8.1 Drug Uptake in 2D

Cellular uptake was assessed by using fluorescence microscopy. MCF-7 and MDA-MB-231 breast cancer cell lines were cultured at a density of 4 × 10^3^ per well on a coverslip within a Petri dish (Fluorodish, World Precision Instruments, Hitchin, U.K.). Following a 48 h incubation period, a 1 μM solution of TMPyP4 (Sigma-Aldrich, Dorset, U.K.) was incubated for another 24 h. After that, TMPyP4 was removed, and the cells were washed with PBS (Gibco, U.K.). The cells were then subjected to a 1 h incubation with 100 nM of Lysotracker Green (Invitrogen, U.K.) and 100 nM of BioTracker 405 Blue Mitochondria Dye (EMD Millipore, Merck, U.K.) prior to imaging. Fluorescence images were subsequently obtained by using a Leica SP8 confocal microscope with a 25× objective. The excitation wavelength used for detecting TMPyP4 was 405 nm, with emission between 650 and 700 nm. For imaging the mitochondria, the excitation wavelength was set to 405 nm, with emission captured between 420 and 450 nm. Lysosomes were imaged by using an excitation wavelength of 488 nm and an emission range of 510–540 nm.

#### 2.8.2 Drug Efficacy in 2D and PDT Treatment

For PDT, a water-soluble porphyrin photosensitizer was employed, TMPyP4 (meso-Tetra­(*N*-methyl-4-pyridyl) porphine tetra tosylate), where the tosylate serves solely as the counterion (Sigma-Aldrich, Dorset, U.K.). To evaluate the cytotoxic effects, MCF-7, MDA-MB-231, HUVECs, MSCs, and HNLF were cultured in a 96-well plate at a density of 4 × 10^3^ cells per well for 48 h. This was followed by a further 48 h incubation with increasing concentrations of TMPyP4, ranging from 0.1 to 4 μM. The cells were washed twice with PBS, and fresh phenol-free cell culture media was subsequently added. For the illumination of cells treated with TMPyP4, a filtered xenon-arc lamp was utilized, featuring a light guide to filter out ultraviolet and infrared emissions (Paterson Lamp, Phototherapeutics Ltd., U.K.). This lamp emitted light within a wavelength range of 420 ± 20 nm at a fluence rate of 6 mW/cm^2^. The irradiation lasted for 5 min, resulting in a cumulative light dose of 1.8 J/cm^2^ (calculated as total light dose (J/cm^2^) = fluence rate (W/cm^2^) × treatment time (s)).^25^ Furthermore, experiments were conducted in which samples were incubated with TMPyP4 in the absence of light (dark conditions) to compare the cytotoxic effects of both treatments. After 24 h, cell viability was evaluated using the CellTiter-Glo 3D Viability Assay.

### 2.9 Experiments in 3D

#### 2.9.1 Drug Uptake in 3D

In order to assess the uptake of TMPyP4 in 3D tumoroids of MCF-7 and MDA-MB-231, 5 μM TMPyP4 was added on the 18th day after seeding, for a period of 48 h. Following this incubation period, fluorescence imaging was conducted with a confocal Leica SP8 inverted microscope, using a 10× objective and an excitation wavelength of 405 nm, with an emission range set between 650 and 700 nm.

#### 2.9.2 Drug Efficacy in 3D

Compartmentalized 3D tumoroids of MCF-7 and MDA-MB-231 were engineered, incorporating two distinct stromal compartments that simulate primary breast cancer and metastatic breast cancer in the lungs. On the 18th day postseeding, the 3D compartmentalized tumoroids were divided into 4 groups as presented below.

a. Control: No treatment (just cell culture media).
b. Photosensitizer-treated: received 5 μM of TMPyP4.
c. Doxorubicin-treated: received 10 μM of doxorubicin.
d. Photosensitizer + Doxorubicin-treated: 5 μM of TMPyP4 + 10 μM of doxorubicin

Following a 48 h incubation period, the drugs were washed twice with PBS, after which fresh phenol-free cell culture media was added. The irradiation protocol described earlier for the 2D culture experiments (light dose of 1.8 J/cm^2^) was also applied to the 3D complex tumoroids. Additionally, experiments were carried out in which samples were incubated with drugs without undergoing irradiation (dark condition). The samples were then allowed to incubate for an additional 24 h postirradiation or no irradiation before the endpoint experiments were conducted. Therapeutic efficacy was assessed via CellTiter Glo 3D Viability Assay, live-dead fluorescence imaging, vascular network complexity, and real-time monitoring of oxygen gradient levels, which are previously described.

To evaluate the potential synergistic effects of doxorubicin and TMPyP4, we employed the following equation to compute the α (α) value^26^

1 $$\frac{\alpha =F_{\mathrm{PDT}}\times F_{\mathrm{cytotoxin}}}{F_{\mathrm{combination}}}$$

In this equation, F_PDT_ and F_cytotoxin_ in the numerator represent the fractional cell viability obtained from each individual treatment. The denominator represents the fractional cell viability resulting from combination treatment. An α value greater than 1 implies a synergistic effect, a value of 1 corresponds to an additive effect, and a value less than 1 indicates an antagonistic effect.^26^

### 2.10 Statistical Analysis

Analysis of the data was carried out using GraphPad Prism 9 software (GraphPad, San Diego, CA). The data were initially tested for normality using the Shapiro-Wilk test. It was determined that two-way ANOVA was justified because multiple parameters were compared.^26^ The two key independent categorical variables, which were simultaneously in play, were the different treatment groups and the light/dark conditions. These combination variables were then used to study their effect on 3 separate outcome variables when subjected to the drug intervention. These included invasion, cell death, and subsequent hypoxia gradient formation. A Two-way ANOVA test of significance was applied to determine if there was an interaction effect between the two factors.^26^ A significance threshold of *p* < 0.05 was used. Data points are presented as means with standard error of the mean (SEM). All experiments were conducted with a minimum of 4 repeats.

## 3 Results

### 3.1 Uptake of Photosensitizer in 2D and 3D

MCF-7 cells take up TMPyP4 more readily than MDA-MB-231 cells in 2D demonstrated by increased red fluorescence inside cells (Figure 2). Photosensitizers are known to accumulate in different organelles, significantly influencing the activation of specific signaling pathways during PDT. To investigate the subcellular distribution of TMPyP4, fluorescent probes LysoTracker (green) and Mitotracker (blue) were used to label the lysosomes and mitochondria. TMPyP4 was present in both lysosomes and mitochondria, though it appeared to cluster more in the mitochondria (Figure 2). Fluorescence imaging of 3D tumoroids incubated with 5 μM of TMPyP4 confirmed that MCF-7 cells in 3D tumoroids demonstrated a greater uptake of TMPyP4 compared to MDA-MB-231 (Figure 3).

**Figure 2:**
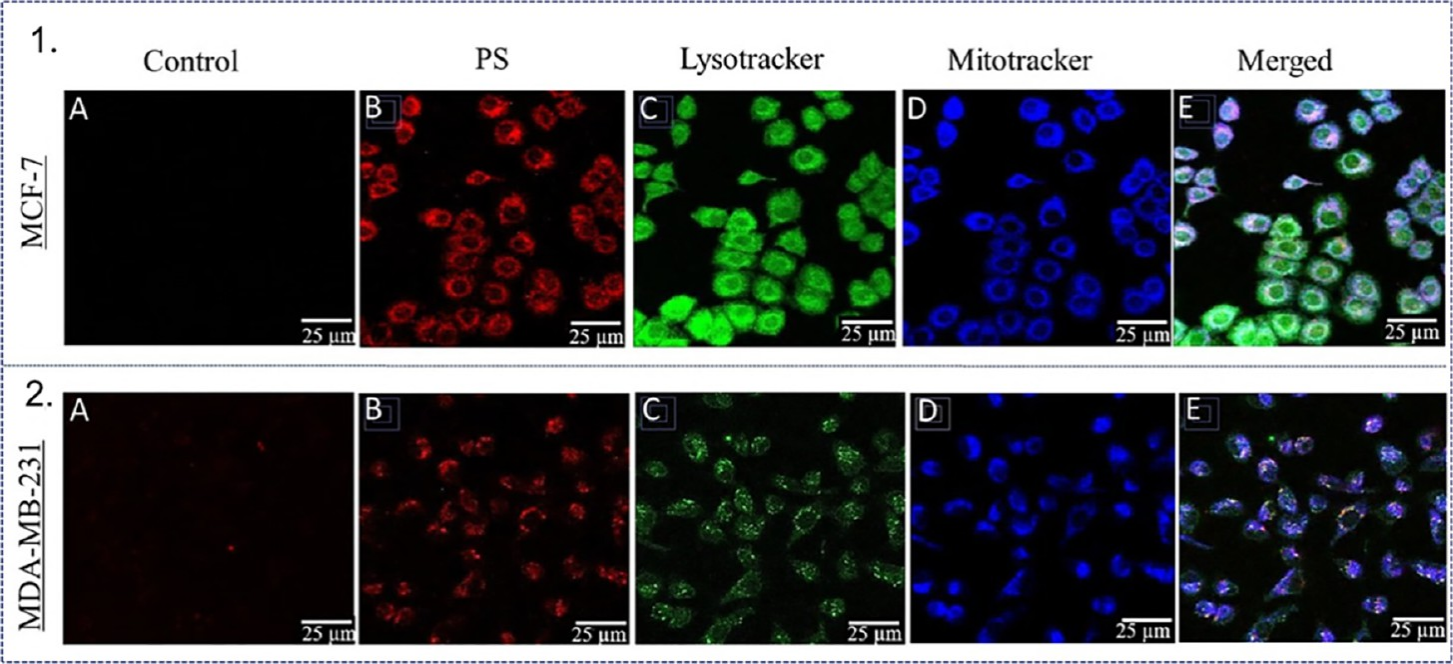
Photosensitizer uptake and localization in 2D cultured breast cancer cells using fluorescence microscopy. (1) Fluorescence images of MCF-7 cells, where A represents control, B represents MCF-7 cells showing red fluorescence of photosensitizer, C represents MCF-7 cells showing green fluorescence of lysosomes, D represents MCF-7 cells showing blue fluorescence of mitochondria, and E represents merged red, green, and blue channels. (2) Fluorescence images of MDA-MB-231 cells, where A represents control, B represents MDA-MB-231 cells showing red fluorescence of photosensitizer, C represents MDA-MB-231 cells showing green fluorescence of lysosomes, D represents MDA-MB-231 cells showing blue fluorescence of mitochondria, and E represents merged red, green, and blue channels. PS: Photosensitizer, TMPyP4 denoted as PS. Scale bar 25 μm. Magnification 25×.

**Figure 3:**
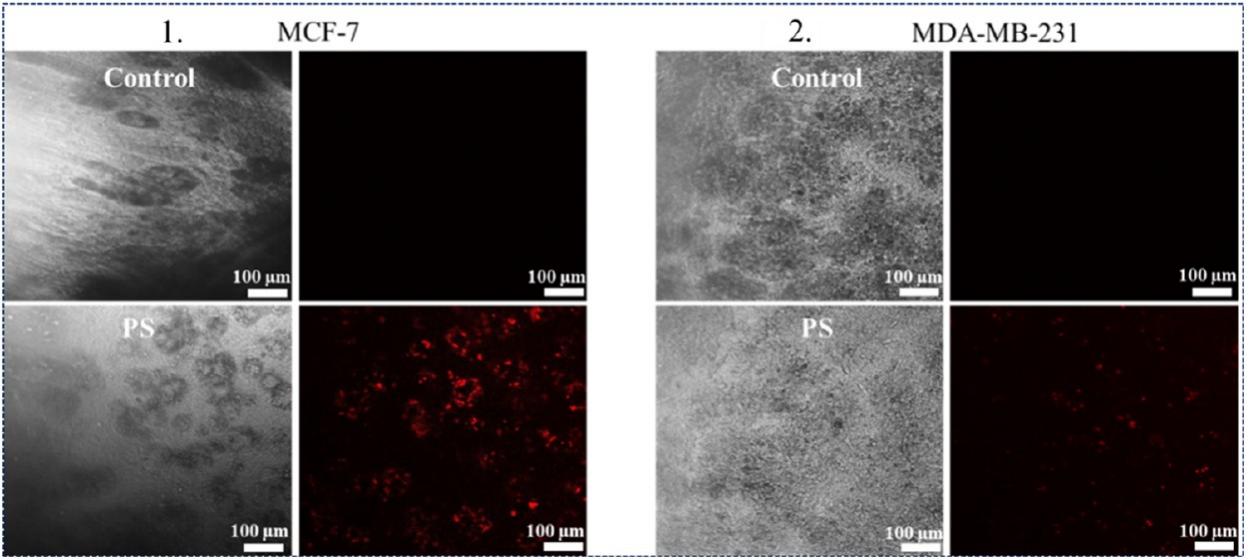
Photosensitizer cellular uptake in 3D cultured breast tumouroids using fluorescence microscopy. Panel 1. shows phase contrast and fluorescence images of MCF-7 TM in 3D breast tumouroids where a bright red fluorescence indicates higher cellular uptake of photosensitizer, and panel 2. shows phase contrast and fluorescence images of MDA-MB-231 TM in 3D breast tumouroids where a minimal red fluorescence of photosensitizer can be observed. TM: Tumor mass, PS: Photosensitizer. TMPyP4 denoted as PS. Magnification 10×.

### 3.2 Photodynamic Therapy Efficacy in 2D

Breast cancer cell lines MCF-7 and MDA-MB-231 exhibited a significant concentration-dependent cytotoxicity when exposed to light-activated TMPyP4, compared to the control and nonactivated TMPyP4. The calculated IC50 for light-activated TMPyP4 was determined to be 0.12 μM for MCF-7 cells and 0.44 μM for MDA-MB-231 cells. Furthermore, light-activated TMPyP4 also induced significant cytotoxicity in mesenchymal stem cells, human lung fibroblasts, and endothelial cells compared to the control and nonactivated TMPyP4. Human lung fibroblasts demonstrated the greatest resistance to light-activated TMPyP4 (IC50 = 0.84 μM) compared to mesenchymal stem cells (IC50 = 0.13 μM) and endothelial cells (IC50 = 0.14 μM) (Figure 4).

**Figure 4:**
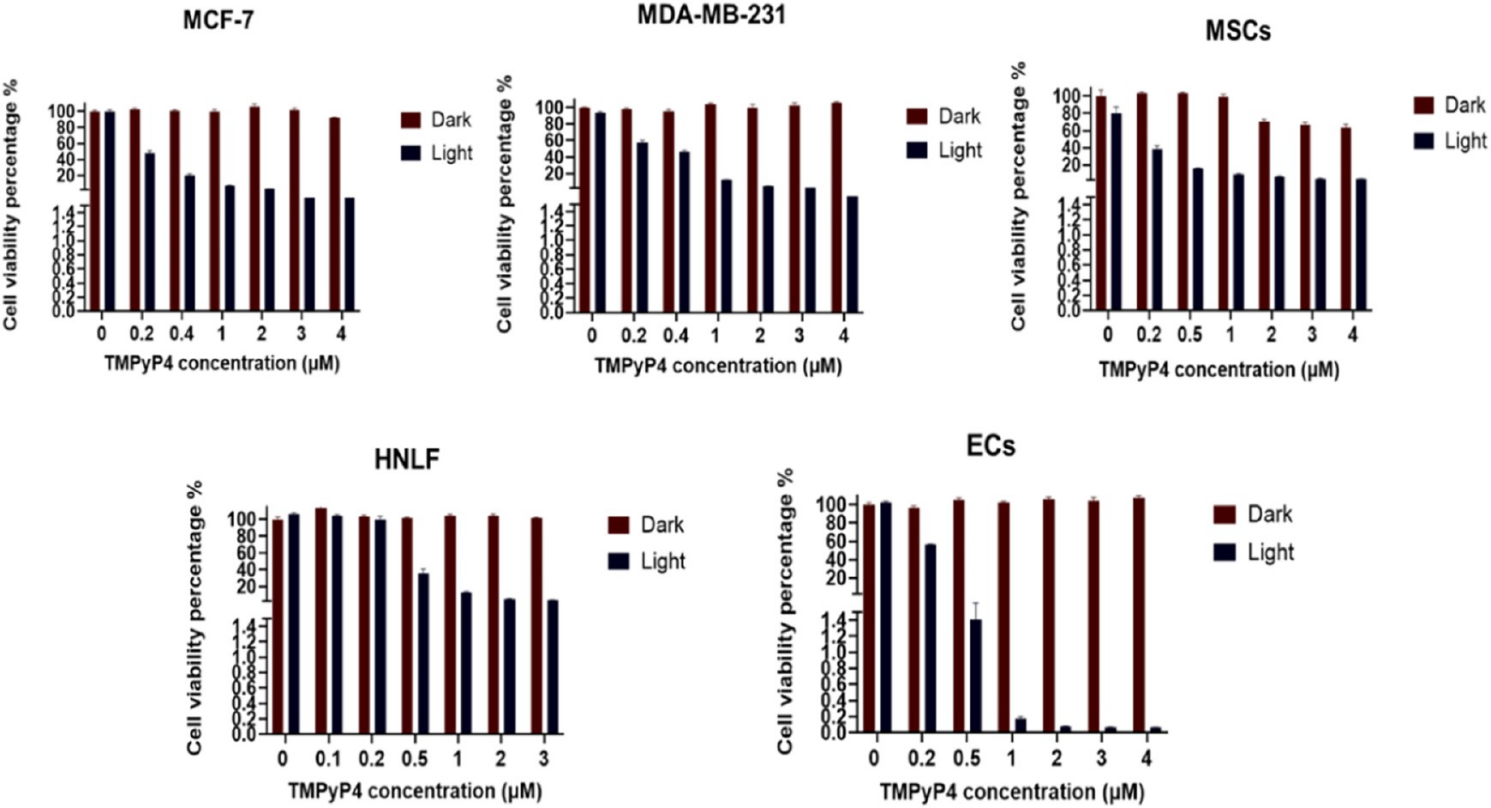
Photosensitizer (TMPyP4) efficacy in 2D cultured breast cancer cells and healthy stromal cells using CellTiter-Glo 3D Viability Assay, shown as graphs of cell viability percentage.

### 3.3 Photodynamic Therapy Efficacy in 3D

#### 3.3.1 Breast Cancer Surrounded by a Primary Stromal Compartment

Light-activated TMPyP4 exhibits a diminished capacity to induce cell death in 3D tumoroids of breast cancer compared to 2D. In MCF-7 3D tumoroids, light-activated TMPyP4 exhibited a statistically significant mean reduction (64.7%) in viable cancer cells within the TM, which corresponds to a 1.5-fold reduction, compared to the controls. The combination of doxorubicin with light-activated TMPyP4 produced a significant mean decline (36.8%) in viability of cancer cells within the TM relative to the control (mean = 98.81%) (*P*-value < 0.0001), light-activated TMPyP4 (mean = 64.7%), and the combination of doxorubicin with nonactivated TMPyP4 (mean = 73.2%) (Figure 5(2)).

**Figure 5:**
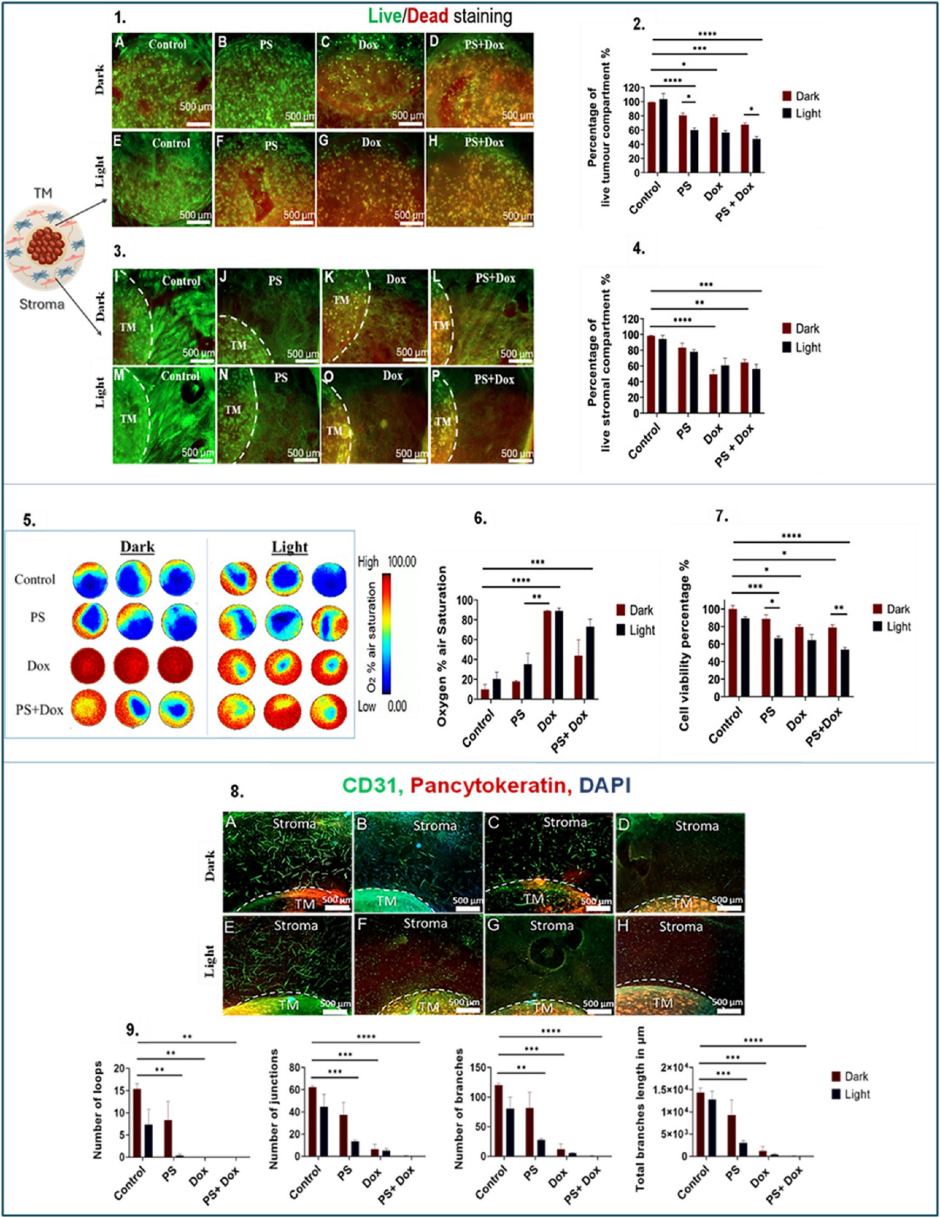
Efficacy of PDT in MCF-7 3D compartmentalized tumoroids with primary stroma. (1) Fluorescence images of the TM, samples (A, dark; E, light) represent control group, (B, dark; F, light) represent samples treated with 5 μM of TMPyP4 denoted as PS, (C, dark; G, light) represent samples treated with 10 μM of doxorubicin, (D, dark; H, light) represent samples treated with combined 5 μM of TMPyP4 and 10 μM of doxorubicin. Treatment with either TMPyP4, doxorubicin, or combination was applied for 48 h. Light-irradiated groups were treated with a blue lamp for 5 min. (2) The corresponding graph of the quantified percentage of live cells; (3) fluorescence images of the stroma, samples (I: dark, M: light) represent control group, samples (J: dark, N: light) represent samples treated with 5 μM of TMPyP4 denoted as PS, samples (K: dark, O: light) represent samples treated with 10 μM of doxorubicin, and samples (L: dark, P: light) represent samples treated with combined 5 μM of TMPyP4 and 10 μM of doxorubicin. (4) The corresponding graph of the quantified percentage of live cells. (5) Pseudocolor images that capture oxygen gradient levels in MCF-7 3D compartmentalized tumoroids. (video in appendix). (6) Graph that shows the recorded percentage of oxygen air saturation. (7) Graph that reflects the percentage of cell viability. (8) Immunofluorescence images of MCF-7 3D compartmentalized tumoroids where panels (A, E), (B, F), (C, G), and (D, H) represent untreated, TMPyP4, doxorubicin, and combined TMPyP4 and doxorubicin-treated groups, respectively. (9) Graph of quantified parameters of vascular network complexity. Scale bar = 500 μm. The analysis is based on *n* = 4, with significance determined through Two-way ANOVA. Data is shown in graphs as the mean and standard error of the mean. * < 0.05, ** <0.01, *** < 0.001, **** < 0.0001. PS: Photosensitizer. TM: tumor mass. Magnification 2.5×.

In tumoroids where the stroma is representative of the primary stromal tissue site (breast tissue), MCF-7 cells within the TM exhibited a more pronounced reduction in the percentage of living cells compared to the stromal compartment when treated with light-activated TMPyP4, either alone or in combination with doxorubicin (Figure 5(2 and 4)). Light activation of TMPyP4 produced a notably greater cytotoxicity (*P*-value = 0.01) than the nonactivated TMPyP4, corresponding to the live/dead assay. Light-activated TMPyP4 alone reduced mean cell viability to 66.4%, and when combined with doxorubicin, mean cell viability further dropped to 53.6%. Using the previously mentioned equation, the calculated α value for the combined doxorubicin and light-activated TMPyP4 was found to be 0.8. Moreover, light-activated TMPyP4 increased oxygenation levels (41.1%), though not significantly, over the untreated (9.7%) and nonactivated groups (17.7%). However, the combination of doxorubicin and light-activated TMPyP4 significantly increased oxygen saturation to 73.1% (*P* < 0.0001), indicating alleviation of tumor hypoxia. Findings of the CellTiter Glo assay corresponded well with real-time oxygen saturation reading (Figure 5(5–7)) and demonstrated an inverse dependence, i.e., where cell viability was high, oxygen levels were low, and following drug action that reduced cell viability, oxygen levels increased, and hypoxia gradients were diminished. MCF-7 tumoroids support the formation of endothelial/vascular network by HUVECS in 3D (Figure 5(8)). Significant decreases in vascular networks in the stroma were observed following treatment with light-activated TMPyP4 (3-fold decrease), doxorubicin (9-fold decrease), and doxorubicin with light-activated TMPyP4 combination (40-fold decrease) (Figure 5(9)).

In MDA-MB-231 3D tumoroids, monotherapy with either light-activated TMPyP4 or doxorubicin alone elicited limited efficacy (Figure 6(2)). However, the combination of doxorubicin and light-activated TMPyP4 demonstrated a significant mean decline in the percentage of live cancer cells in the TM (*P*-value = 0.02), from 99% to 71.06%. In the stromal compartment of the MDA-MB-231 3D tumoroids, combining doxorubicin and light-activated TMPyP4 resulted in a significant mean reduction (59.1 %) of viable cells compared to (97.3 %) in the untreated group and (101.4 %) in the light-activated TMPyP4 group, as shown in Figure 6(4).

**Figure 6:**
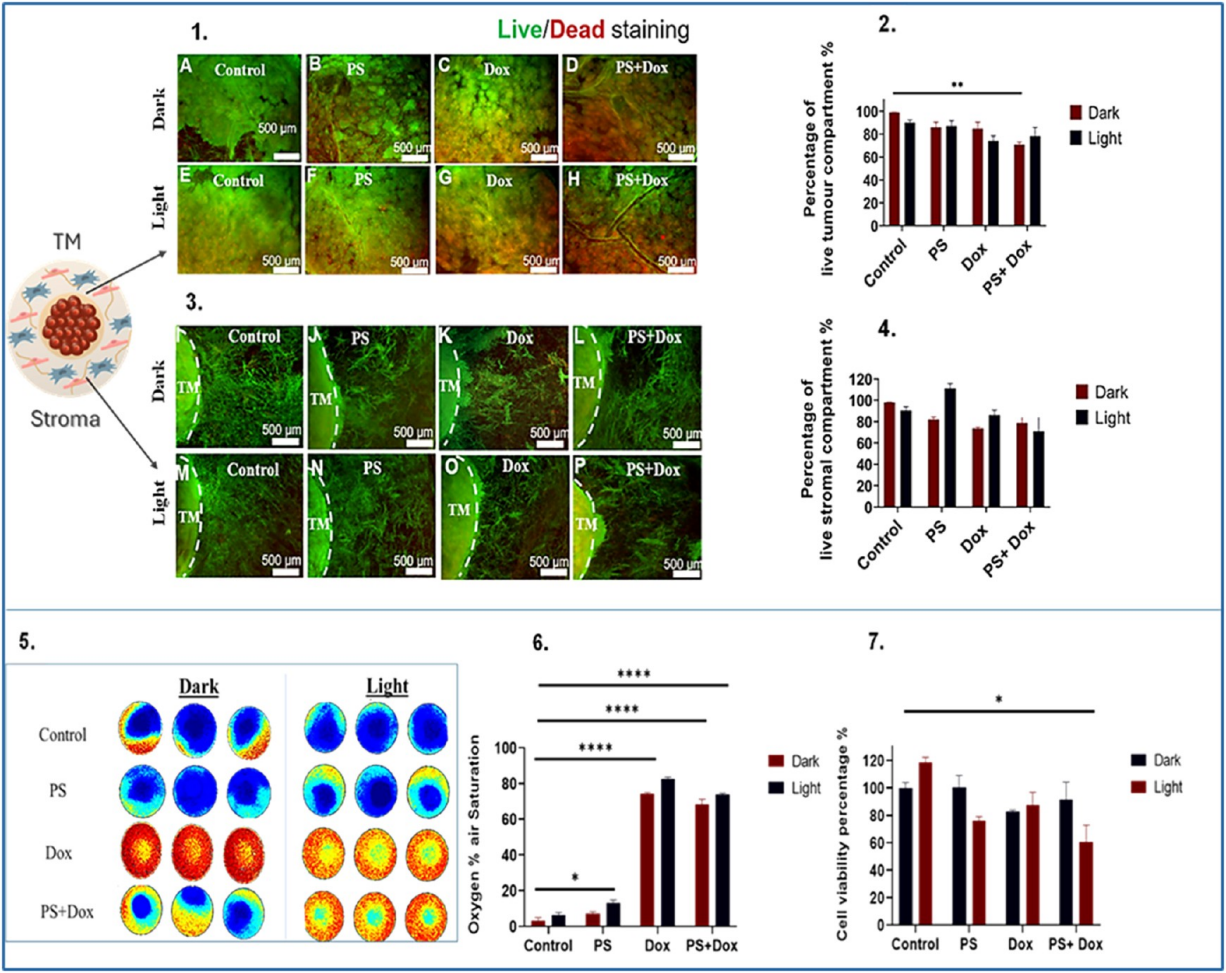
Efficacy of PDT in MDA-MB-231 3D compartmentalised tumouroids with primary stroma. Panel 1. shows fluorescence images of the TM, samples (A: dark, E: light) represent control group, (B: dark, F: light) represent samples treated with 5 μM of TMPyP4 denoted as PS, (C: dark, G: light) represent samples treated with 10 μM of doxorubicin, (D: dark, H: light) represent samples treated with combined 5 μM of TMPyP4 and 10 μM of doxorubicin. Treatment with either TMPyP4, doxorubicin or combination was applied for 48 h. Light irradiatiated groups were treated with a blue lamp for 5 min. Panel 2. shows the corresponding graph of the quantified percentage of live cells, panel 3. shows fluorescence images of the stroma, samples (I: dark, M: light) represent control group, (J: dark, N: light) represent samples treated with 5 μM of TMPyP4 denoted as PS, (K: dark, O: light) represent samples treated with 10 μM of doxorubicin, (L: dark, P: light) represent samples treated with combined 5 μM of TMPyP4 and 10 μM of doxorubicin. Panel 4. shows the corresponding graph of the quantified percentage of live cells. Panel 5. represents pseudocolor images that capture oxygen gradient levels in MDA-MB-231 3D complex tumouroids (video in appendix). Panel 6. represents a graph that shows the recorded percentage of oxygen air saturation. Panel 7. shows a graph that reflects the percentage of cell viability. Scale bar = 500 μm. The analysis is based on *n* = 4, with significance determined through Two-way ANOVA. Data is shown in graphs as the mean and standard error of the mean. * <0.05, ** <0.01, *** <0.001, **** <0.001. PS: Photosensitiser. TM: tumour mass. Magnification 2.5×.

The CellTiter Glo assay demonstrated that the only significant mean decline (60.6%) in cell viability among the different treatment regimens was the combination of doxorubicin and light-activated TMPyP4. MDA-MB-231 3D tumoroids treated with light-activated TMPyP4 or doxorubicin exhibited a nonsignificant reduction in cell viability (Figure 6(7)). The calculated value of α (α = 1.002) indicated that the combination of doxorubicin and light-activated TMPyP4 only produced an additive effect. This contrasts with the MCF-7 calculated value (α = 0.8), reinforcing cell line-specific differences in therapeutic interaction. Real-time monitoring of oxygen levels in MDA-MB-231 3D tumoroids revealed that light-activated TMPyP4 led to a slight improvement in oxygenation (12.9%) compared to the control group (3.2%). It was also found that doxorubicin, in conjunction with light-activated TMPyP4, significantly increased oxygenation levels from 3.2% to 73.8% (*P*-value < 0.0001) (Figure 6(6)). It should be noted that MDA-MB-231 3D tumoroids did not support the formation of vascular networks, thus rendering vascular network analysis redundant.

#### 3.3.2 Breast Cancer Surrounded by a Metastatic Stromal Compartment

In MCF-7 3D tumoroids, which had a metastatic stromal compartment, live-dead imaging revealed that treatment with light-activated TMPyP4 led to a substantial mean reduction (22.4%) in the proportion of viable cancer cells in the TM corresponding to ∼5-fold reduction compared to the control group and the group treated with nonactivated TMPyP4 (89.5%), as shown in (Figure 7(2)). Although the combination of doxorubicin and light-activated TMPyP4 also reduced the percentage of live cancer cells in the TM to 20.7%, no differences were observed between the individual and combination treatment groups. HLFs within the stromal compartment of MCF-7 3D tumoroids were impacted by the application of light-activated TMPyP4 with a mean reduction (9.2%) in viability compared to (100%) in the control group and (87.9 %) in the nonactivated TMPyP4 group. Treatment with doxorubicin resulted in a notable mean decrease (41.4%) in live cells, and the combination of doxorubicin and light-activated TMPyP4 led to a further mean reduction (15%) relative to the control group. It is noteworthy that the group treated solely with light-activated TMPyP4 showed a lower percentage of live cells compared to those treated with either doxorubicin or the combined treatment (Figure 7(2 and 4)).

**Figure 7:**
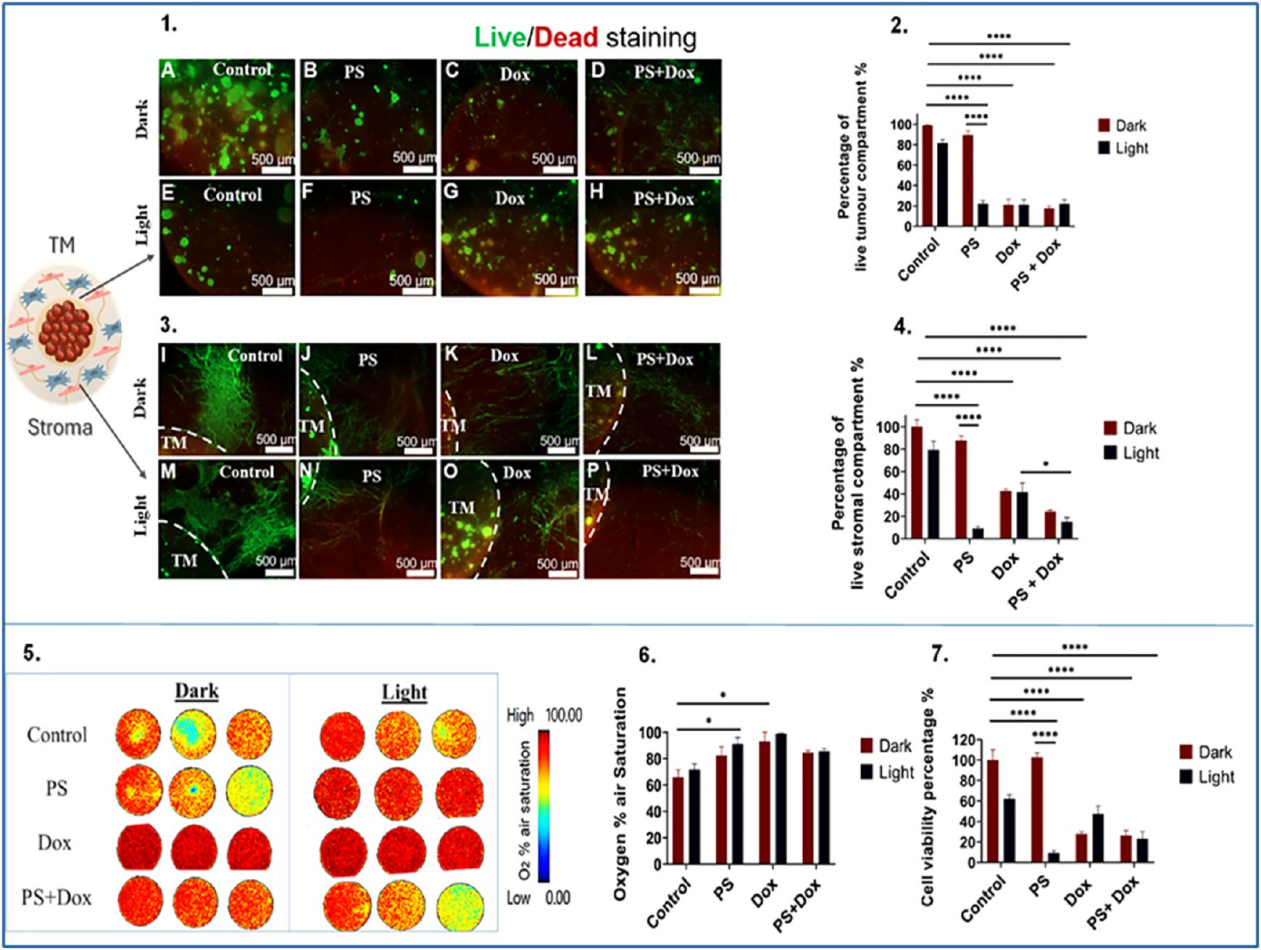
Efficacy of PDT in MCF-7 3D compartmentalized tumoroids with a metastatic stroma. (1) Fluorescence images of the TM, samples (A: dark, E: light) represent control group, (B: dark, F: light) represent samples treated with 5 μM of TMPyP4 denoted as PS, (C: dark, G: light) represent samples treated with 10 μM of doxorubicin, and (D: dark, H: light) represent samples treated with combined 5 μM of TMPyP4 and 10 μM of doxorubicin. Treatment with either TMPyP4, doxorubicin, or combination was applied for 48 hours. Light-irradiated groups were treated with a blue lamp for 5 min. (2) The corresponding graph of the quantified percentage of live cells. (3) Fluorescence images of the stroma, samples (I: dark, M: light) represent control group, (J: dark, N: light) represent samples treated with 5 μM of TMPyP4 denoted as PS, (K: dark, O: light) represent samples treated with 10 μM of doxorubicin, (L: dark, P: light) represent samples treated with combined 5 μM of TMPyP4 and 10 μM of doxorubicin. (4) The corresponding graph of the quantified percentage of live cells. (5) Pseudocolor images that capture oxygen gradient levels in MCF-7 3D compartmentalized tumoroids. (6) Graph showing the recorded percentage of oxygen air saturation. (7) Graph reflecting the percentage of cell viability. Scale bar = 500 μm. The analysis is based on *n* = 4, with significance determined through two-way ANOVA. Data is shown in graphs as the mean and standard error of the mean. * < 0.05, ** <0.01, *** < 0.001, **** < 0.0001. PS: Photosensitizer. TM: tumor mass. Magnification 2.5×.

The CellTiter Glo assay correlated with the live-dead imaging results, with the exception of the control group exposed to light, which had reduced viability (Figure 7(7)) and all treatments resulted in significantly reduced viabilities, most notably for PDT treatment at 9.2 %. The calculated α value indicated that there was no synergistic effect observed between the combinations of doxorubicin and light-activated TMPyP4. Interestingly, real-time oxygen monitoring of MCF-7 3D tumoroids revealed that the baseline oxygen levels in the untreated control group remained relatively stable, unlike those in the MCF-7 3D tumoroids, containing the primary stromal compartment. This denotes that oxygen consumption and metabolic activity in MCF-7 3D tumoroids with primary stroma are higher than those containing the metastatic stroma, which could be attributed to differences in the stromal cells. However, the groups treated solely with doxorubicin and light-activated TMPyP4 demonstrated a statistically significant enhancement in oxygenation levels, with means of 91.05 and 98.9%, respectively, in comparison to the control group (Figure 7(6)).

In the 3D tumoroids of MDA-MB-231, light-activated TMPyP4 resulted in a statistically significant mean decrease (59.5%) in live cancer cells within the TM which corresponds to a 1.7-fold reduction compared to (99.4%) in the control, as shown in (Figure 8(2)). Additionally, doxorubicin alone resulted in a mean survival rate of 62.5%, while the combination of doxorubicin with light-activated TMPyP4 reduced live cell proportions to 55.8%. Nevertheless, no significant differences were found between the groups treated with the combination of doxorubicin and light-activated TMPyP4 and those treated with doxorubicin alone or light-activated TMPyP4 alone. In the stromal compartment of MDA-MB-231 3D tumoroids, the group treated with light-activated TMPyP4 demonstrated a significant mean reduction (57.4%) in viable cells, compared to (100%) in the control group and (99.1%) in the nonactivated TMPyP4 group. Furthermore, both the doxorubicin-treated group and the group receiving a combination of doxorubicin and light-activated TMPyP4 showed significant mean reductions in live cells, with mean values of 62 and 54.2%, respectively, relative to 99.4% in the control group (Figure 8(2 and 4)).

**Figure 8:**
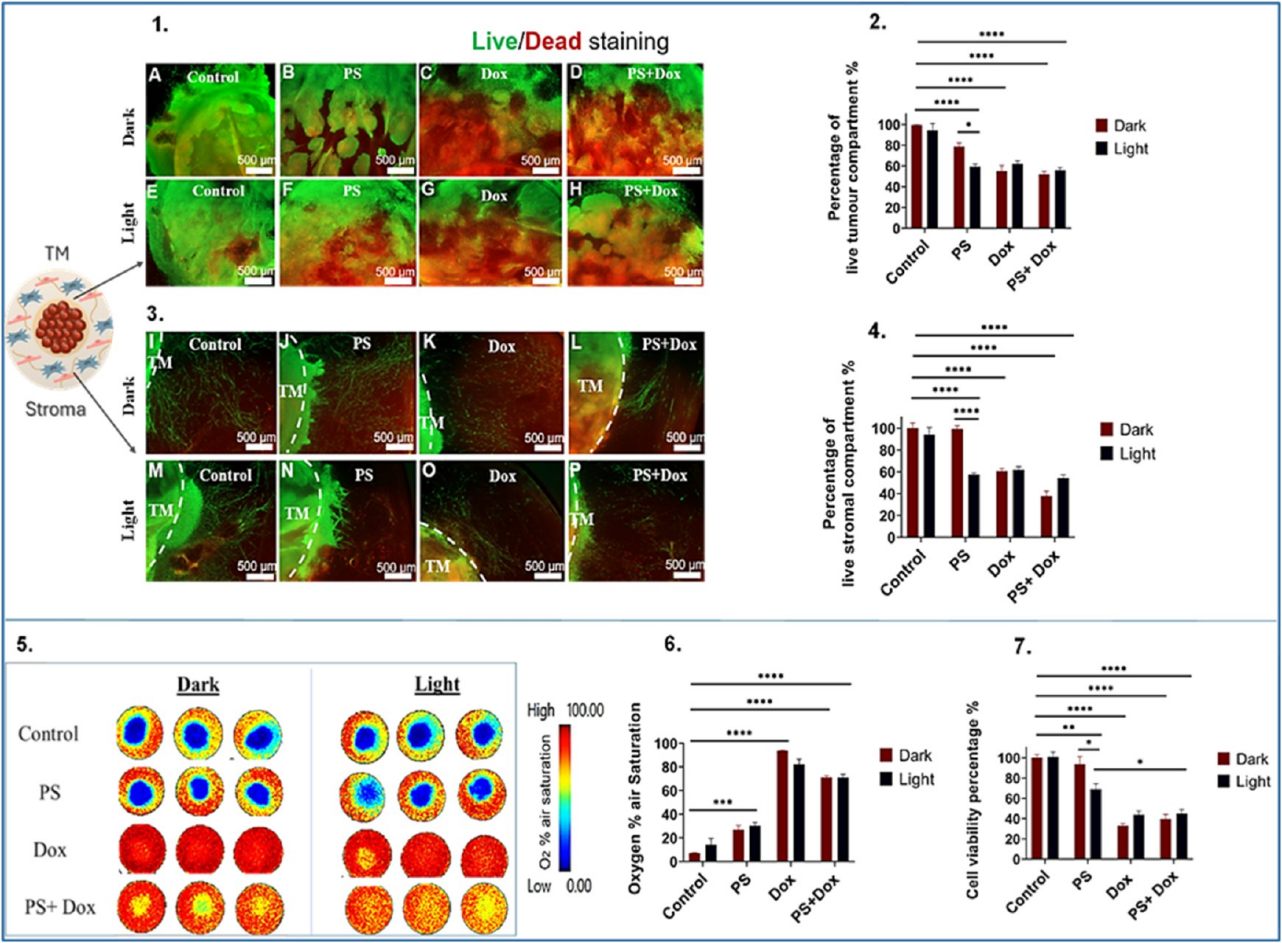
Efficacy of photodynamic therapy in MDA-MB-231 3D compartmentalized tumoroids with a metastatic stroma. (1) Fluorescence images of the TM, samples (A: dark, E: light) represent control group, (B: dark, F: light) represent samples treated with 5 μM of TMPyP4 denoted as PS, (C: dark, G: light) represent samples treated with 10 μM of doxorubicin, and (D: dark, H: light) represent samples treated with combined 5 μM of TMPyP4 and 10 μM of doxorubicin. (2) The corresponding graph of the quantified percentage of live cells. (3) Fluorescence images of the stroma, samples (I: dark, M: light) represent control group, (J: dark, N: light) represent samples treated with 5 μM of TMPyP4 denoted as PS, (K: dark, O: light) represent samples treated with 10 μM of doxorubicin, and (L: dark, P: light) represent samples treated with combined 5 μM of TMPyP4 and 10 μM of doxorubicin. (4) The corresponding graph of the quantified percentage of live cells. (5) Pseudocolor images that capture oxygen gradient levels in MDA-MB-231 3D compartmentalized tumoroids. (6) Graph showing the recorded percentage of oxygen air saturation. (7) Graph reflecting the percentage of cell viability. Scale bar = 500 μm. The analysis is based on *n* = 4, with significance determined through two-way ANOVA. Data is shown in graphs as the mean and standard error of the mean. * < 0.05, ** <0.01, *** < 0.001, **** < 0.0001. PS: Photosensitizer. TM: tumor mass. Magnification 2.5×.

According to the CellTiter Glo assay, light-activated TMPyP4 treatment induced a significant mean decline of 69.5% in cell viability, as shown in Figure 8(7), which corresponds to a 1.5-fold reduction compared to the control group (99.9%). The mean viabilities observed for the primary vs metastatic stroma tumoroids were comparable at 76.1 and 69.5% respectively, corresponding to a 1.1-fold reduction in viability for the metastatic stroma. In contrast to the MCF-7 studies, a markedly larger difference was observed for primary (66.4%) vs metastatic stroma (9.2%), corresponding to a 7.2-fold reduction in viability. Treatment of the MDA-MB-231 tumoroids with doxorubicin, as well as the combination of doxorubicin and light-activated TMPyP4 resulted in markedly lower percentages of viable cells, averaging 43.8 and 44.9%, respectively. The calculated α value showed that there was no synergistic effect evident on combining doxorubicin and light-activated TMPyP4 (Figure 8(7)).

Real-time oxygen monitoring of MDA-MB-231 tumoroids, revealed a statistically significant enhancement in oxygenation levels within the light-activated TMPyP4-treated group, reaching an average of 30.3% compared to the control group (7.3%). Furthermore, the combination of doxorubicin and light-activated TMPyP4 demonstrated a remarkable improvement (*P*-value < 0.0001) in oxygenation levels, reaching a mean of 71.1% air oxygen saturation, although this was lower than the doxorubicin-only group, which reached 82.2% air oxygen saturation (Figure 8(6)). Both MCF-7 and MDA-MB-231 3D tumoroids with metastatic stroma showed the absence of vascular networks formation.

## 4 Discussion

This study explored the therapeutic efficacy of PDT both as an independent treatment modality and in combination with the chemotherapeutic agent doxorubicin utilizing an advanced multicompartment 3D tumoroid model of breast cancer. The tumoroid model comprises a central TM which is surrounded by a stromal compartment incorporating a dense matrix prepared via plastic compression. A comparative study was carried out using two human breast carcinoma cell lines with either a primary breast stromal compartment or a metastatic lung stroma compartment.

A previous study also described the development of multicompartment breast tumor organoids featuring two main ECM compartments: the basement membrane composed of Matrigel and the stromal matrix composed of collagen type I.^27^ Additionally, researchers incorporated primary monocytes isolated from PBMCs or immortalized macrophage U87 cell lines into the stroma. However, our tumoroids developed herein incorporated various stromal cells including adipose tissue-derived MSCs and HNLF in addition to endothelial cells, which are critical components of the TME. Also, a variety of extracellular matrix proteins such as laminin and fibrin in addition to collagen type I were utilized in our model. Moreover, the plastic compression method employed herein increases the stiffness of the ECM, which improves the biomimicry of our model to physiological in vivo tissue stiffness. The photosensitizer employed in this study is TMPyP4, which is a cationic porphyrin characterized by a porphyrin ring that incorporates four *N*-methyl-4-pyridyl substituents. TMPyP4 displays multiple absorption peaks, with its most significant peak occurring at 424 nm in the blue region of the visible spectrum, which corresponds to the Soret band.^28^ Its fluorescence emission is characterized by two prominent peaks within the red spectral region, at 649–655 nm and 715–720 nm.^28,29^ TMPyP4 was selected in this study due to its known efficacy as a photosensitizer in PDT. Its advantageous properties include water solubility, effective permeability across cellular membranes, and a notable quantum yield of singlet oxygen (^1^O_2_) (Φ = 0.77 ± 0.04).^30,31^ Furthermore, TMPyP4 has shown the ability to intercalate into and stabilize G-quadruplex structures within DNA,^32^ leading to the inhibition of telomerase activity.^33^

PDT exerts its primary anticancer effects through direct destruction of tumor cells via apoptosis, necrosis, or autophagy,^16^ provocation of an inflammatory response recruitment of leukocytes to the affected area, and activation of antitumor T-lymphocytes.^34^ Furthermore, PDT treatment may compromise the tumor microvasculature, resulting in diminished oxygen and nutrient supply that is vital for tumor viability.^35^ ROS inflict damage on vascular ECs, triggering clotting processes, promoting platelet aggregation, and leading to vascular obstruction. This vascular occlusion results in sustained hypoxia within tumor tissue, ultimately culminating in cell death.^35^ However, it is important to note that this effect may also intensify tumor hypoxia, potentially leading to the development of resistance to PDT.

Studies were carried out initially in standard 2D culture and simple tumoroid models without a stromal component. MCF-7 cancer cells exhibit a greater uptake of TMPyP4 in both 2D and 3D cultures when compared to MDA-MB-231 cells (Figures 2 and 3). Electrostatic attraction between positively charged TMPyP4 and negative cell membrane likely drives its cellular uptake. However, our results contradict the expectation that TMPyP4 uptake would be greater in MDA-MB-231 cells, which are reported to be more negatively charged than MCF-7 cells.^36,37^ Evidence suggests that clathrin-mediated endocytosis is particularly efficient for positively charged (cationic) molecules due to their strong interaction with negatively charged cell membranes.^38–40^ Given that MCF-7 cancer cells predominantly rely on the clathrin-mediated endocytosis mechanism,^41–43^ this may explain the observed higher uptake of TMPyP4 in MCF-7 cells in this study.

TMPyP4 at 1 μM incubated for 24 h predominantly localizes within the mitochondria with a smaller presence in lysosomes in both MCF-7 and MDA-MB-231 cells in 2D (Figure 2), which may be attributed to the elevated transmembrane potential of the inner mitochondrial membrane, that facilitates the uptake of cationic molecules into the mitochondrial matrix.^44^ One study has shown nuclear localization of 10 μM TMPyP4 in MCF-7 cells after 3 h of incubation,^44^ while another study reported cytoplasmic localization of 1 μM TMPyP4 in MCF-7 cells following 12 h of incubation^45^ which concurs with our findings. Additionally, one study has shown that prior to illumination, TMPyP4 was primarily confined to the lysosomes of colon carcinoma cells; however, following irradiation, the photosensitizer was relocated to the nucleus and nucleoli.^46^

2D cultures of MCF-7 and MDA-MB-231 cancer cells treated with TMPyP4 PDT exhibited significant cytotoxicity in a concentration-dependent manner (Figure 4). Notably, MCF-7 cells demonstrated greater sensitivity to the cytotoxic effects of TMPyP4 PDT than MDA-MB-231. Several studies have documented the anticancer effects of TMPyP4 through telomerase inhibition in breast cancer cells, particularly at concentrations of 5 μM or higher.^33,47,48^ Additionally, other research investigated its effectiveness as a photosensitizer for PDT in breast cancer.^44,49^ Interestingly, our findings also indicate that approximately six times higher TMPyP4 concentration is required to kill 50% of the HNLF lung fibroblasts compared to MSCs. In contrast, ECs were found to be the most sensitive to the cytotoxic effects of light-activated TMPyP4. Aligned with other studies,^29,49^ data presented here suggest that normal lung fibroblasts may be more resistant to the effects of PDT.

PDT efficacy was investigated using 3D compartmentalized tumoroids incorporating both TM and a stromal compartment mimicking *in vivo* tumors. PDT studies using a variety of 3D models have provided valuable insight into a range of therapeutic variables, including the development of immune responses.^50,51^ In order to replicate primary breast cancer effectively, a stromal compartment containing adipose tissue-derived MSCs and ECs was engineered alongside TM in dense collagen to mimic physiological collagen densities and restrict oxygen diffusion.^23^ PDT and doxorubicin are both oxygen-dependent treatments, and thus, the hypoxia gradient mimicry within tumoroids is a necessary feature for physiological relevance. 5 μM TMPyP4 concentration was selected based on preliminary experiments performed with 3D simple tumoroids of just the TM (Supporting 2). These preliminary experiments revealed a lower drug susceptibility for cells in 3D models compared to 2D models, and therefore, a higher TMPyP4 concentration was needed, as reported previously by Hadi et al. using a disulfonated porphyrin photosensitizer.^26^ The concentration of doxorubicin used herein was based on a study by Pradhan et al.^52^

Monotherapy with light-activated TMPyP4 in MCF-7 3D compartmentalized tumoroids surrounded by primary stromal compartment produced significant cytotoxicity, especially within the TM (Figure 5). This correlated to a moderate enhancement of oxygen levels and a significant disruption of the vascular networks (Figure 5). The increase in oxygenation levels following PDT treatment is interpreted as resulting from the increased level of cytotoxicity, which leads to reduced oxygen consumption within the construct owing to cellular respiration. PDT can also induce oxygen consumption during treatment via photochemical reactions, such as the reaction of singlet oxygen with cellular substrates. In these experiments, however, the oxygen measurements were carried out 48 h post-treatment; therefore, the measurements should primarily reflect the changes in cell viability induced by the treatment.

Unlike the MCF-7 studies, treatment of MDA-MB-231 3D compartmentalized tumoroids with light-activated TMPyP4 monotherapy proved less effective. Combination treatment of MDA-MB-231 tumoroids with doxorubicin and light-activated TMPyP4 did elicit stronger cytotoxicity, albeit merely an additive effect, as manifested by a substantial decrease in cell viability within both the TM and the stromal compartment (Figure 6). In summary, for models with a primary stroma compartment, the findings show that only the MCF-7 3D tumoroids exhibited a significant response to the cytotoxic effects of PDT monotherapy, eliciting a pronounced impact on the TM (*P*-value < 0.0001) compared to the adjacent stromal compartment (*P*-value = 0.01). In contrast, using combination therapy, treatment of MDA-MB-231 3D tumoroids with PDT and doxorubicin elicited a significant cell killing in the TM compartment (*P*-value = 0.02). The reduced efficacy of light-activated TMPyP4 treatment in MDA-MB-231 3D compartmentalized tumoroids could be explained by the lower uptake of TMPyP4 noted in 3D tumoroids (Figure 3). Hypoxic tumors present a huge obstacle that compromises the effectiveness of PDT, and MDA-MB-231 TMs exhibit significantly greater hypoxia relative to MCF-7 TM (Figures 5 and 6), which may render them more resistant to PDT.

Several studies have shown that combining chemotherapeutic agents with PDT can enhance the breast cancer treatment efficacy. In MDA-MB-231 cells, treatment with 5-aminolevulinic acid PDT in conjunction with cisplatin caused a marked reduction in cell viability compared to either therapy alone.^53^ Co-administration of TMPyP/phthalocyanine photosensitizers alongside doxorubicin facilitated death in breast cancer cells *in vivo*.^54^ Similarly, light-activated chlorin-vitamin conjugates and their respective indium complexes, combined with Taxol, exhibited a pronounced synergistic cytotoxicity against TNBC cell lines.^55^ A different combination therapy with pheophorbide A PDT and doxorubicin showed mainly additive effects in HeLa cells, although in one case, a synergistic effect was observed. The aforementioned study was carried out in 2D systems, but in our 3D system where doxorubicin was administered before PDT was carried out, the effect of combination treatment was additive rather than synergistic. While doxorubicin can generate singlet oxygen upon light activation, this is largely suppressed when doxorubicin is bound to DNA in the nucleus.^56^ Herein, we did not observe any significant differences between the light and dark cytotoxicity levels with doxorubicin monotherapy. The effect of combined PDT and doxorubicin treatment in MDA-MB-231 3D compartmentalized tumoroids observed herein may arise from complementary mechanisms, as doxorubicin primarily targets nuclear DNA, whereas TMPyP4 can photodamage lysosomes or mitochondria (Figure 2), facilitating the activation of separate pro-apoptotic signaling pathways.^57–59^ Thus, PDT can effectively bypass several key regulatory points associated with resistance to chemotherapy. Additionally, light activation of TMPyP4 may induce oxidative damage to the efflux pumps responsible for expelling doxorubicin from the cells, increasing its intracellular concentration.^60,61^ PDT effectively restores the sensitivity of adriamycin-resistant breast cancer cells to adriamycin via inhibition of MDR1 gene expression.^62^ In the present study, however, PDT was applied after administration of doxorubicin; therefore, PDT impairment of efflux pumps may exert a relatively minor effect on overall efficacy.

The treatment efficacy of both PDT and doxorubicin in a 3D compartmentalized tumoroid model of metastatic breast cancer, containing HNLFs and ECs, was significantly enhanced (Figures 7 and 8), in comparison to tumoroids with a primary stromal component. However, no synergistic effects were detected from the combination treatment. The lung fibroblasts used in the metastatic model were relatively resistant to PDT when cultured in 2D (Figure 4). The superior efficacy of doxorubicin or light-activated TMPyP4 treatment observed in this modified model may result from ECM differences between 3D compartmentalized tumoroids with metastatic and primary breast stroma. Existing evidence indicates that ECM composition, density, stiffness, and mechanical properties can vary significantly between primary breast tumors and metastatic lesions in the lungs. Within the TME, the ECM serves not only as structural support but also as an active regulator of tumor progression.^63^ ECM components incorporated in our model included collagen type I, laminin, and fibrin. Increased collagen cross-linking (via LOX enzymes) and fiber alignment enhance mechanical tension, activating signaling pathways that drive tumor cell proliferation, invasion, and drug resistance.^64,65^ Laminin and fibrin support angiogenesis and provide a scaffold for migrating tumor cells.^66–68^ Nevertheless, it is important to consider that ECM remodeling (ECM deposition and degradation) is a continuous dynamic process that occurs throughout tumorigenesis. Another point to consider in our 3D compartmentalized models is that ECM remodeling differs markedly between stroma containing MSCs and HNLFs. A recent study investigated ECM remodeling *in vivo* within the premetastatic lungs of mice harboring primary breast tumor xenografts. Lung fibroblasts demonstrated an increase in the levels of collagen (Col) Col4A5, MMP2, MMP3, and MMP14, associated with reduced levels of LOX, LOXL2, and prolyl 4-hydroxylase α-1.^69^ Additionally evidence of elastin degradation alongside an upregulation of MMP-2, MMP-4, and MMP-9 expression within the pulmonary tissue of a murine model for breast cancer was observed during the third and fourth weeks.^70^ Also, stromal cell function and activity can be crucially reprogrammed by tumor cells such as conversion of fibroblasts into CAFs which show altered expression of MMPs and LOX enzymes, reflecting ECM reorganization^71–73^ or transformation of adipose tissue-derived stem cells into CAFs.^74^ A change in the ECM stiffness in 3D compartmentalized tumoroids featuring metastatic stroma could potentially influence drug penetration, tumor oxygenation, and the matrix-driven signaling pathways in cancer cells. ECM stiffness critically modulates breast cancer biology, driving cell proliferation, stemness, metastasis, and invasion^75^ through key signaling pathways such as YAP/TAZ,^76^ Wnt/β-catenin, FAK,^77^ TWIST1,^78^ PI3K/AKT/mTORC1,^79^ and ROCK.^80^ Consequently, alterations in matrix stiffness can profoundly influence these signaling cascades, thereby affecting the behavior of breast cancer cells, the functions of stromal cells, and responses to therapeutic interventions. Models similar to this have shown that the tumor cell transcriptome is altered depending on the interaction with different stromal cells, highlighting the key role the TME has on tumor progression.^81^ Furthermore, stromal cells within the TME could distinctly affect the response to therapeutic agents in cases of primary breast cancer compared to metastatic breast cancer. Karimnia et al. demonstrated that photodestruction of stromal fibroblasts in a 3D coculture with PDAC cell lines enhanced tumor response to PDT.^82^ Additionally, under hypoxic conditions, typical of dense 3D matrices as demonstrated herein, hypoxia-inducible factors (HIF-1α and HIF-2α) in cancer cells orchestrate broad genetic programs that reprogram stromal cells via the expression of TGF-β, IL-1, PDGF, and bFGF, promoting the differentiation of normal fibroblasts into CAFs. Hypoxia modulates tumor-infiltrating immune cells,^83^ enhances the recruitment of immune-tolerant cells^84^ and directs the transcriptional activation of immunosuppressive factors which has been shown to reduce the activity of effector cells such as cytotoxic T lymphocytes (CTLs), NK cells, and DC cells.^85^ At the same time, hypoxia upregulates immunosuppressive regulatory T cells, MDSCs, and TAMs, stimulates the secretion of immune-suppressing cytokines and chemokines.^86,87^

While this study provides insights into the influence of ECM composition and stromal context on therapeutic response in 3D breast cancer tumoroids, certain limitations should be acknowledged to guide future work. The current model lacks immune and functional vascular components, which restricts the ability to fully assess tumor–immune interactions and drug delivery dynamics observed *in vivo*. The ECM proteins incorporated in the 3D model do not encompass the full biochemical complexity of the native tumor tissue.

### 4.1 Conclusion and Future Directions

This study utilized a 3D breast tumor-stroma model to investigate the efficacy of PDT and chemotherapy. A differential response of distinct breast cancer subtypes to PDT treatment was demonstrated by enhanced efficacy in MCF-7 tumoroids in contrast to MDA-MB-231 tumoroids. Moreover, since the model incorporates a central tumor mass (TM) surrounded by a stromal component, we were able to identify differential responses to treatment between the TM and the stromal components.

The study also explored the impact of different 3D stromal tissues on the treatment efficacy, which, to the best of our knowledge, has not been previously explored in PDT. A comparative analysis was conducted between breast cancer models featuring either a primary breast stromal compartment or a metastatic lung stroma compartment. For 3D tumoroids with a metastatic stroma, treatment with either a photosensitizer or doxorubicin alone demonstrated significant cytotoxic effects when compared to models incorporating a primary breast stroma.

We examined the development of hypoxia in the 3D constructs and the response to treatment using real-time oxygen monitoring, since the presence of hypoxia is an important factor in determining treatment efficacy for a range of reasons. These studies generated an intriguing and potentially useful finding in that we were able to demonstrate that oxygenation levels measured post-treatment correlated with the treatment-induced cytotoxicity, which indicates that hypoxia gradients can act as a surrogate measurement for treatment efficacy. This technique has not been employed previously for studying PDT or combination therapy, and our results suggest that it could prove of value to future mechanistic studies.

In summary, we found that alterations within the TME significantly influence the efficacy of PDT, as shown by the higher effectiveness of PDT-TMPyP4 as a standalone therapeutic agent within the 3D breast cancer model featuring a metastatic stromal compartment, as opposed to the model with a primary stromal compartment.

The outcomes of this study emphasize the importance of a personalized approach to breast cancer therapy, advocating for the adoption of therapeutic strategies based on the specific subtypes of the disease, as well as the location, whether it is a primary or metastatic site. Future research can involve evaluating the effectiveness of PDT within such 3D models that incorporate patient-derived tissues, including both cancer cells and stromal cells, such as cancer-associated fibroblasts, tumor-associated macrophages, and tumor-associated endothelial cells from both primary and metastatic sites. 3D models that utilize a dense collagen matrix should also be useful for nanoparticle studies involving PDT since diffusion through the matrix is a limiting factor for nanoparticle delivery. Moreover, combining nanodelivery systems with strategies for the mitigation of hypoxia in tumor tissues, modification of the TME through normalization of tumor vasculature and ECM, and inhibition of hypoxia-related proteins could prove beneficial.

## Supplementary Material



## Notes

Fluorescence imaging depicting characteristics of the 3D tumoroids (Figure S1) and therapeutic efficacy of PDT in simple tumoroids (tumor mass only) using live-dead fluorescence imaging (Figure S2) (PDF)
