# Supplementary material for: Engineering Complex Breast Tumor-Stroma Models: TMPyP4-Photodynamic Therapy Is More Effective at the Metastatic Site in Breast Tumors
Source: ACS Biomater Sci Eng. 2025 Dec 29;12(1):515–30. doi: 10.1021/acsbiomaterials.5c01341 (PMC12801187; doi:10.1021/acsbiomaterials.5c01341)
Supplement: Supplementary file 1 [file ab5c01341_si_001.pdf]

Engineering Complex Breast Tumour-Stroma Models: TMPyP4-Photodynamic Therapy Is More Effective At The Metastatic Site In Breast Tumours

Salma T. Rafik [1, 2], Jasmine Ho[1], Alexander J. MacRobert [1], Umber Cheema [1*]

[1]UCL Centre for 3D Models of Health and Disease, UCL Division of Surgery and Interventional Science, Faculty of Medical Sciences, Charles Bell House, 43-45 Foley Street, University College London, London, W1W 7TY, UK.

[2]Department of Clinical Pharmacology, Faculty of Medicine, Alexandria University, Alexandria 21516, Egypt.

*** Correspondence:** salma.galal.20@ucl.ac.uk

u.cheema@ucl.ac.uk

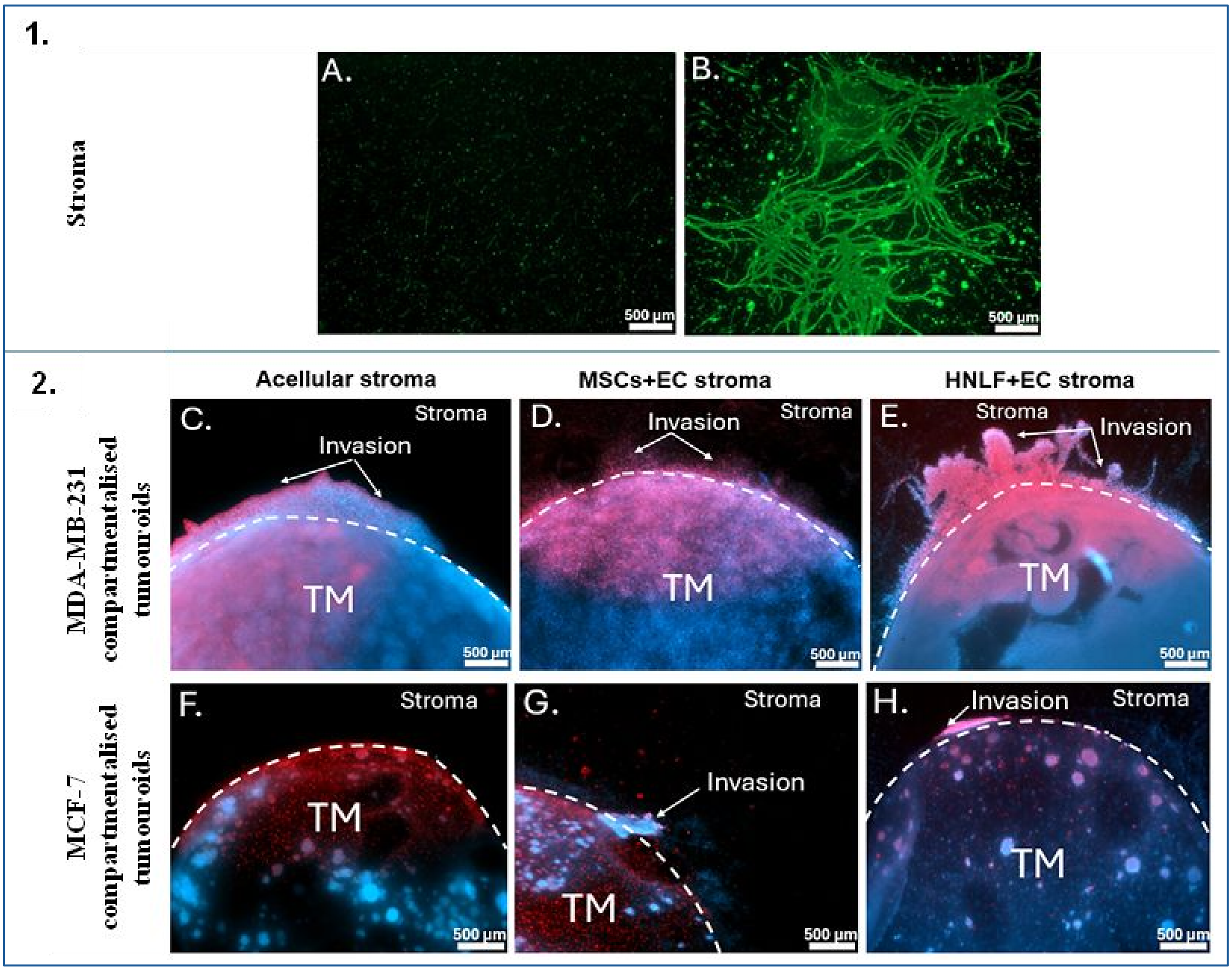

Figure S1: Fluorescence imaging depicting characteristics of the 3D tumouroids. 1A. shows absence of vascular network formation in the 3D constructs of the stromal compartment containing HNLF and ECs, while **1B.** shows well-formed vascular networks in the 3D constructs of the stromal compartment containing MSCs and ECs. **2C.** shows cancer invasion in MDA-MB-231 3D tumouroids with acellular stroma. **2D.** shows less cancer invasion in MDA-MB-231 3D tumouroids with a stromal compartment comprising MSCs and ECs. **2E.** shows greater cancer invasion in MDA-MB-231 3D tumouroids with a stromal compartment comprising HNLF and ECs. **2F.** shows absence of cancer invasion in MCF-7 3D tumouroids with acellular stroma. **2G.** shows some cancer invasion in MCF-7 3D tumouroids with a stromal compartment comprising MSCs and ECs. **2H.** shows slightly more cancer invasion in MCF-7 3D tumouroids with a stromal compartment comprising HNLF and ECs. TM: Tumour mass. Green: CD31, Red: Pancytokeratin and Blue: DAPI. Scale bar 500 µm. Magnification 2.5x.

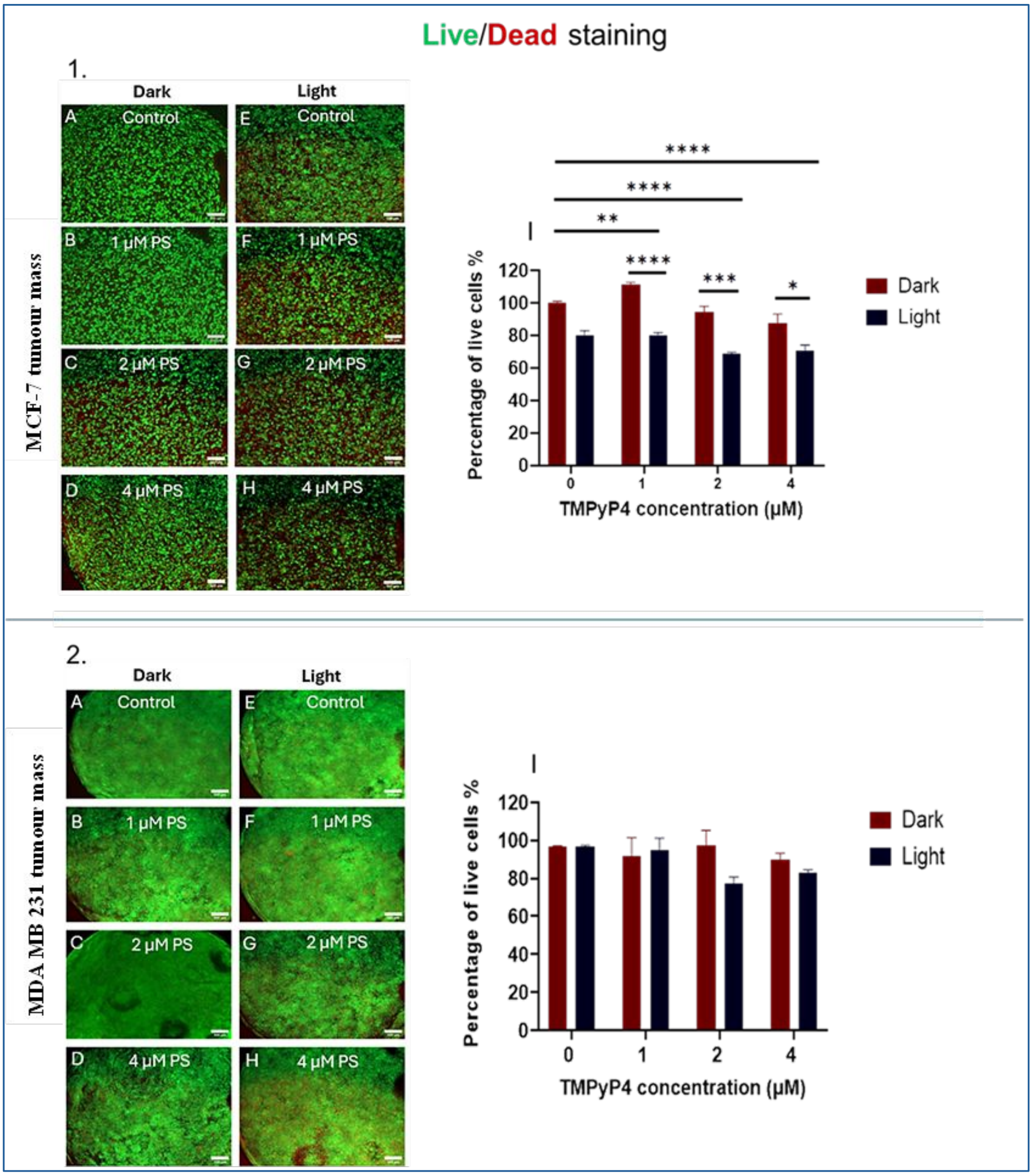

Figure S2: Therapeutic efficacy of PDT in simple tumouroids (Tumour mass only) using live-dead fluorescence imaging. The samples were incubated with either 1, 2 or 4 µM of photosensitiser for 48 hours, followed by blue light illumination for 5 minutes. Live-dead staining was utilised for the assessment of the efficacy of PDT. **Panel 1.** shows fluorescence images of MCF-7 tumour mass only samples where (A, E) represent control samples, (B, F) represent samples treated with 1 µM of photosensitiser, (C, G) represent samples treated with 2 µM of photosensitiser and (D, H) represent samples treated with 4 µM of photosensitiser. Graph I shows the corresponding plot of the quantified percentage of live cells vs. PS concentration. **Panel 2.** shows fluorescence images of MDA-MB-231 tumour mass only samples where (A, E) represent control samples, (B, F) represent samples treated with 1 µM of photosensitiser, (C, G) represent samples treated with 2 µM of photosensitiser and (D, H) represent samples treated with 4 µM of photosensitiser. Graph I shows the corresponding plot of the quantified percentage of live cells vs. PS concentration. PS: Photosensitiser. Scale bar 500 µm. Magnification 2.5x.
